# Supplementary figures and images for: Reversal of neuronal tau pathology, metabolic dysfunction, and electrophysiological defects via adiponectin pathway-dependent AMPK activation
Source: bioRxiv. 2024 Feb 7:2024.02.07.579204. Preprint. [Version 1] doi: 10.1101/2024.02.07.579204 (PMC10871331; doi:10.1101/2024.02.07.579204)

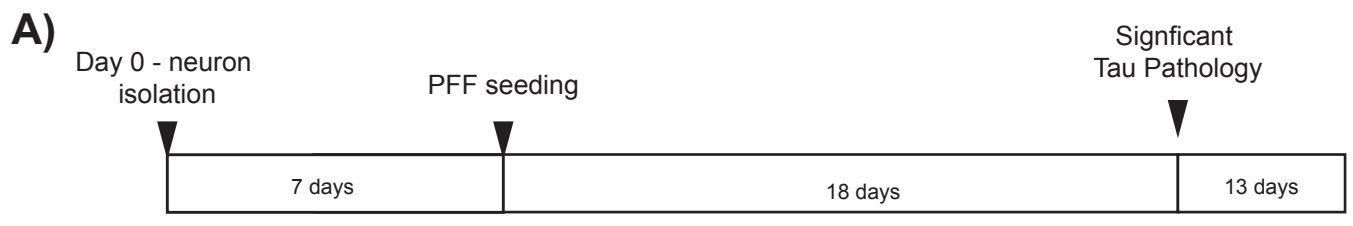

**B)**

AdipoRon treatment and assessment

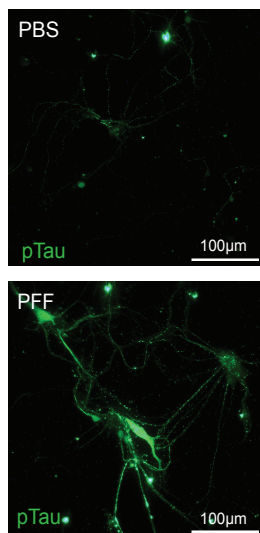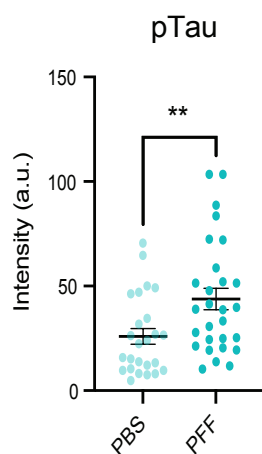

**C)**

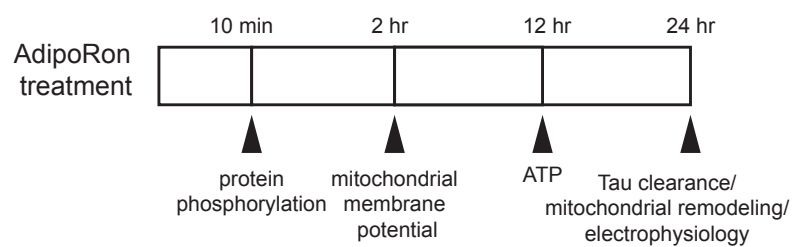

Supplement: Supplement 1 — Figure S1: A) Schematic of neuron culture timeline. Neurons were isolated from postnatal day 0 mice. Neurons were seeded with 3 μg/mL PFF at day in vitro (DIV) 7. Cultures were maintained until at least DIV 25 before experimentation. B) Immunofluorescence of phosphorylated tau (AT-8) in Tau neurons seeded with PFF seeded or PBS control (n=25–28 cells) (scale bar 100 μm). Data shown as mean ± SEM. Significance determined by Student’s t-test with Welch’s correction. C) Schematic of AdipoRon treatment times used in this paper. Protein phosphorylation events were measured after 10-minute treatment. Mitochondrial membrane potential was assessed 2 hours after treatment. Tau clearance, mitochondrial morphology, neuronal architecture, and electrophysiology were measured after 24-hour treatment. [file media-1.pdf]

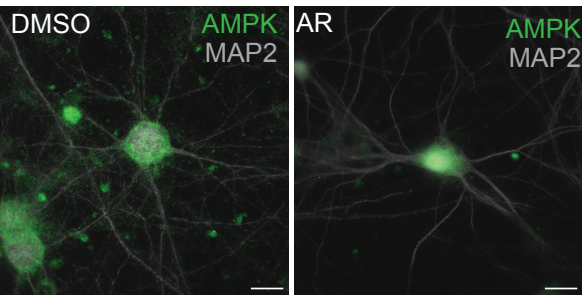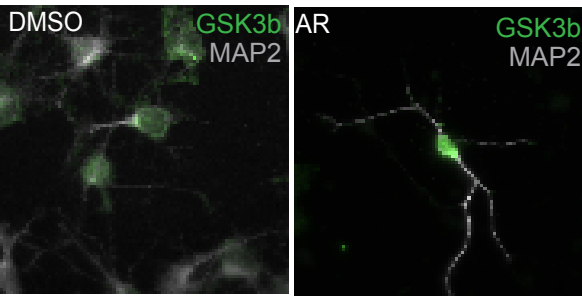

Supplement: Supplement 2 — Figure S2: Representative immunofluorescence images of total protein levels of AMPK and GSK3b. Quantification is shown in Fig 1. [file media-2.pdf]

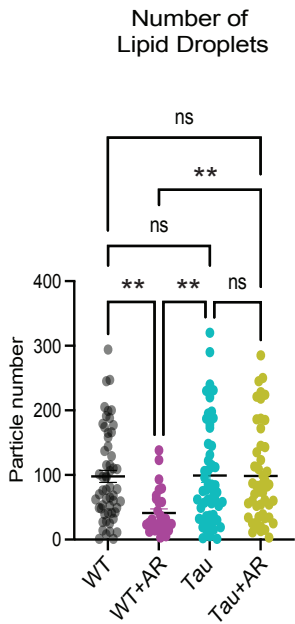

Pyrimidine Metabolism

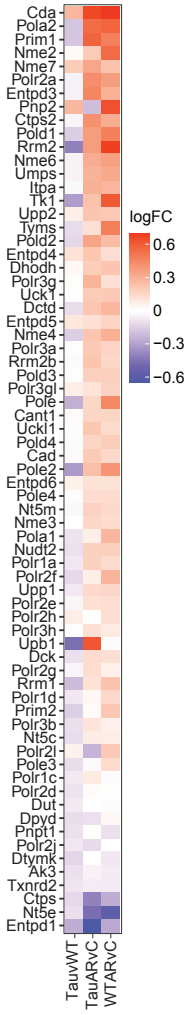

Amino/ Nucleotide Sugar Metabolism

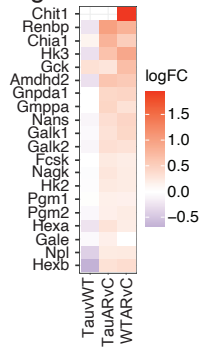

Supplement: Supplement 4 — Fig S4: Quantification of lipid droplet number from neurons shown in Fig.3I. Data shown as mean ± SEM. Heatmaps of Pyrimidine metabolism and amino/ nucleotide sugar metabolism GSEA pathways in AR-treated WT and Tau neurons. [file media-4.pdf]

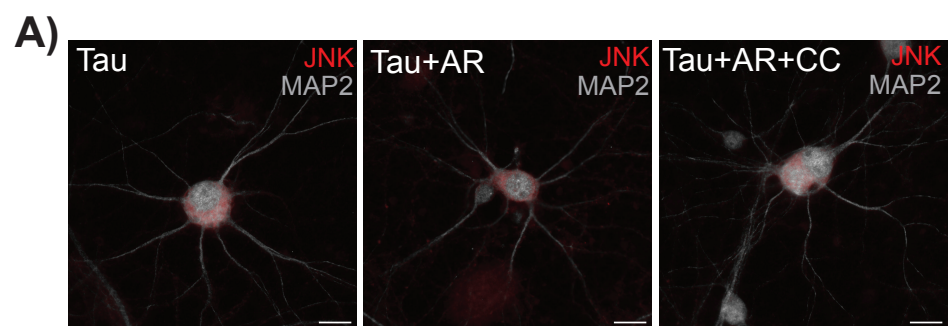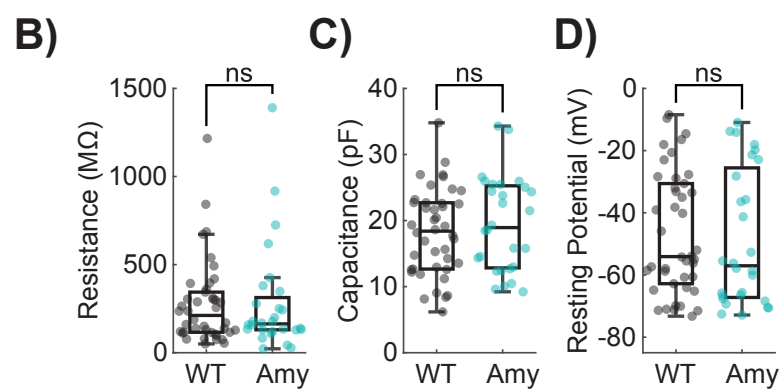

Supplement: Supplement 5 — Fig S5. A) Representative immunofluorescence images of total protein levels of JNK. Quantification shown in Fig.4D. B–D) Resistance (B), capacitance (C), and resting potential (D) for APP/PS1 and WT neurons. Box plots display the median, interquartile range, and whiskers extending to values within the interquartile range multiplied by 1.5. Statistics were performed using Mann–Whitney U tests. D) Spike counts measured in response to stepwise current applications of 20 pA from −100–480 pA for each cell from rest (left) and from −60 mV (right). Statistics were performed using repeated measures two-way ANOVA with Geisser–Greenhouse correction and post-hoc Tukey tests. Error bars are SEM. [file media-5.pdf]

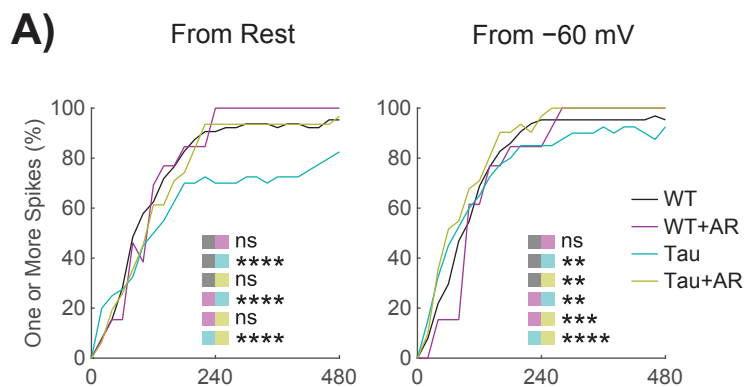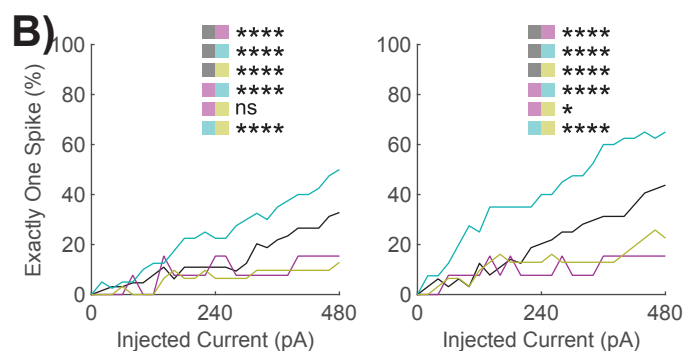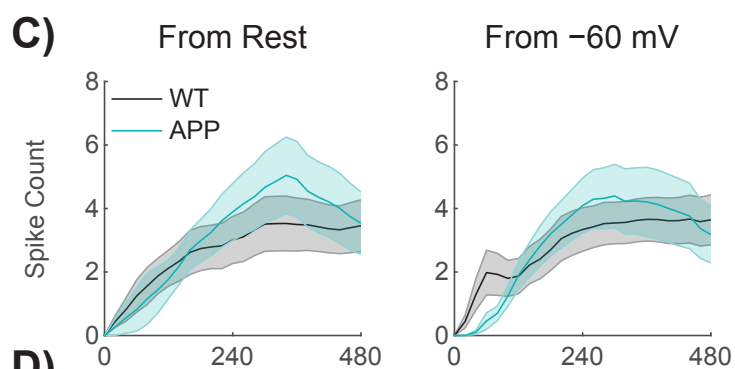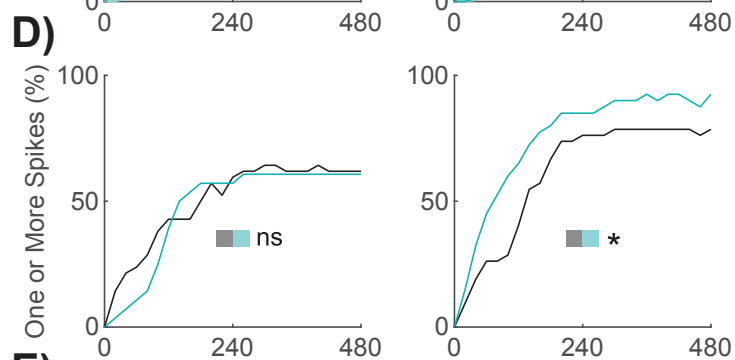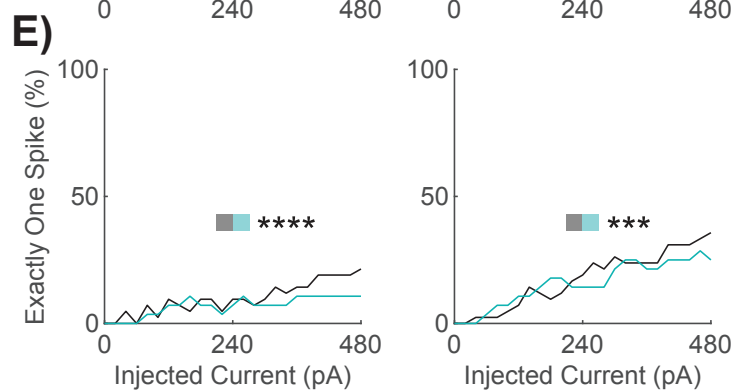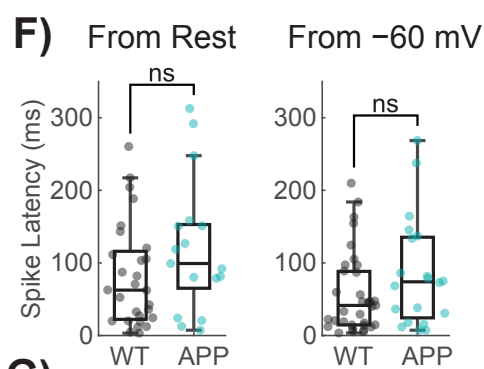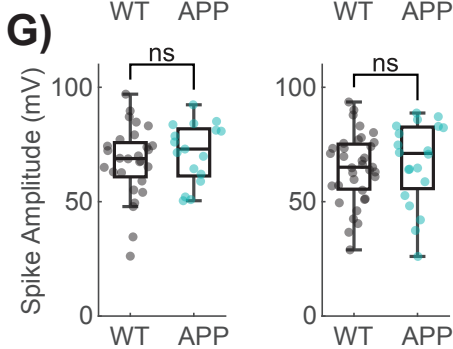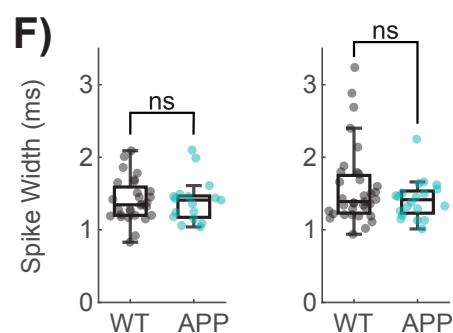

Supplement: Supplement 6 — Fig S6: The percentage of cells firing one or more spikes (A) or exactly one spike (B) was measured across current steps. Lines were compared in a pairwise manner using F-tests of a one-phase association curve passing through the origin for one or more spike plots, and a line through the origin for the exactly one spike plots. Bonferroni corrections were applied to account for the multiple pairwise comparisons. Significance between groups is displayed as paired colored blocks. Sample sizes are: WT: 64, WT+AR: 13, Tau: 40, Tau+AR: 31. C) Action potential spike counts for APP/PS1 and WT neurons were measured in response to 30 stepwise current applications of 20 pA from −100–480 pA for each cell from rest (left) and from −60 mV (right). Statistics were performed using repeated measures two-way ANOVA with Geisser–Greenhouse correction and post-hoc Tukey tests. D-E) The percentage of cells firing one or more spikes (D) or exactly one spike (E) was measured across current steps. Lines were compared in a pairwise manner using F-tests of a one-phase association curve passing through the origin for one or more spike plots, and a line through the origin for the exactly one spike plots. Significance between groups is displayed as paired colored blocks. F-H) Spike latency (F), amplitude (G), and width (H) for the first spike fired at rheobase were collected from current-clamp protocols performed for each cell from rest (left) and from −60 mV (right). Spike width was measured from the full width at half amplitude. Box plots display the median, interquartile range, and whiskers extending to values within the interquartile range multiplied by 1.5. Statistics were performed using Mann–Whitney U tests. Error bars are SEM. Sample sizes for A–F and J–L are WT: 42, Amy: 28. Sample sizes for G–I are WT: 28, Amy: 17 from rest and WT: 35, Amy: 20 from −60 mV. (* p<0.05, *** p<0.001, **** p<0.0001). [file media-6.pdf]

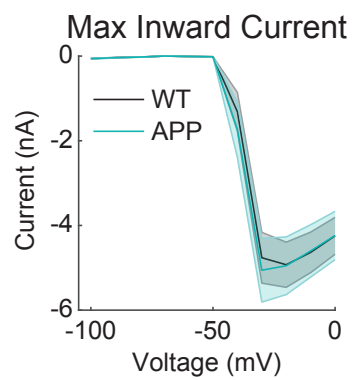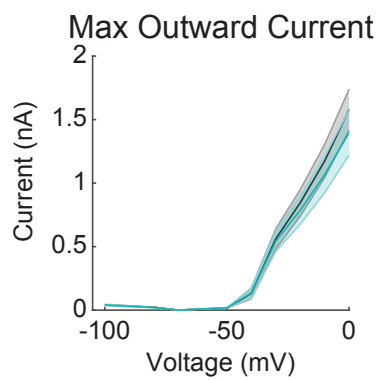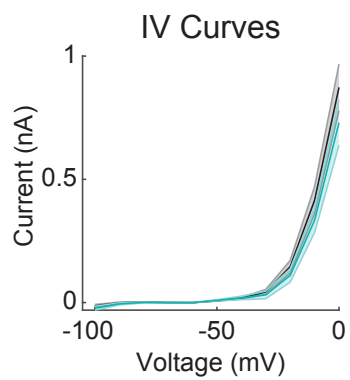

Supplement: Supplement 7 — Fig. S7: The maximum inward current, outward current,and IV curves in APP/PS1 and WT neurons in response to stepwise voltage injections of 10 mV from 0–100 mV. Statistics were performed using repeated measures two-way ANOVA with Geisser–Greenhouse correction and post-hoc Tukey tests. Error bars are SEM. Sample sizes for A–F and J–L are WT: 42, Amy: 28. Sample sizes for G–I are WT: 28, Amy: 17 from rest and WT: 35, Amy: 20 from −60 mV. [file media-7.pdf]
