# Supplementary material for: Reversal of neuronal tau pathology, metabolic dysfunction, and electrophysiological defects via adiponectin pathway-dependent AMPK activation
Source: bioRxiv. 2024 Feb 7:2024.02.07.579204. Preprint. [Version 1] doi: 10.1101/2024.02.07.579204 (PMC10871331; doi:10.1101/2024.02.07.579204)
Supplement: Supplement 3 — Fig S3: A) Volcano plots displaying the differentially expressed genes in RNAseq data. Comparisons shown are Tau vs. WT neurons, Tau neurons AR vs. Control, and WT neurons AR vs. Control. B) Venn diagram showing overlap between differentially expressed genes in the 3 comparisons shown in A. C) Venn diagram showing the overlap between enriched KEGG pathways in the 3 comparisons shown in A. D) Complete STRING diagram from the Neuroactive Ligand Receptor interaction pathway. This diagram was curated to show only the large networks in Fig.2C. [file media-3.pdf]

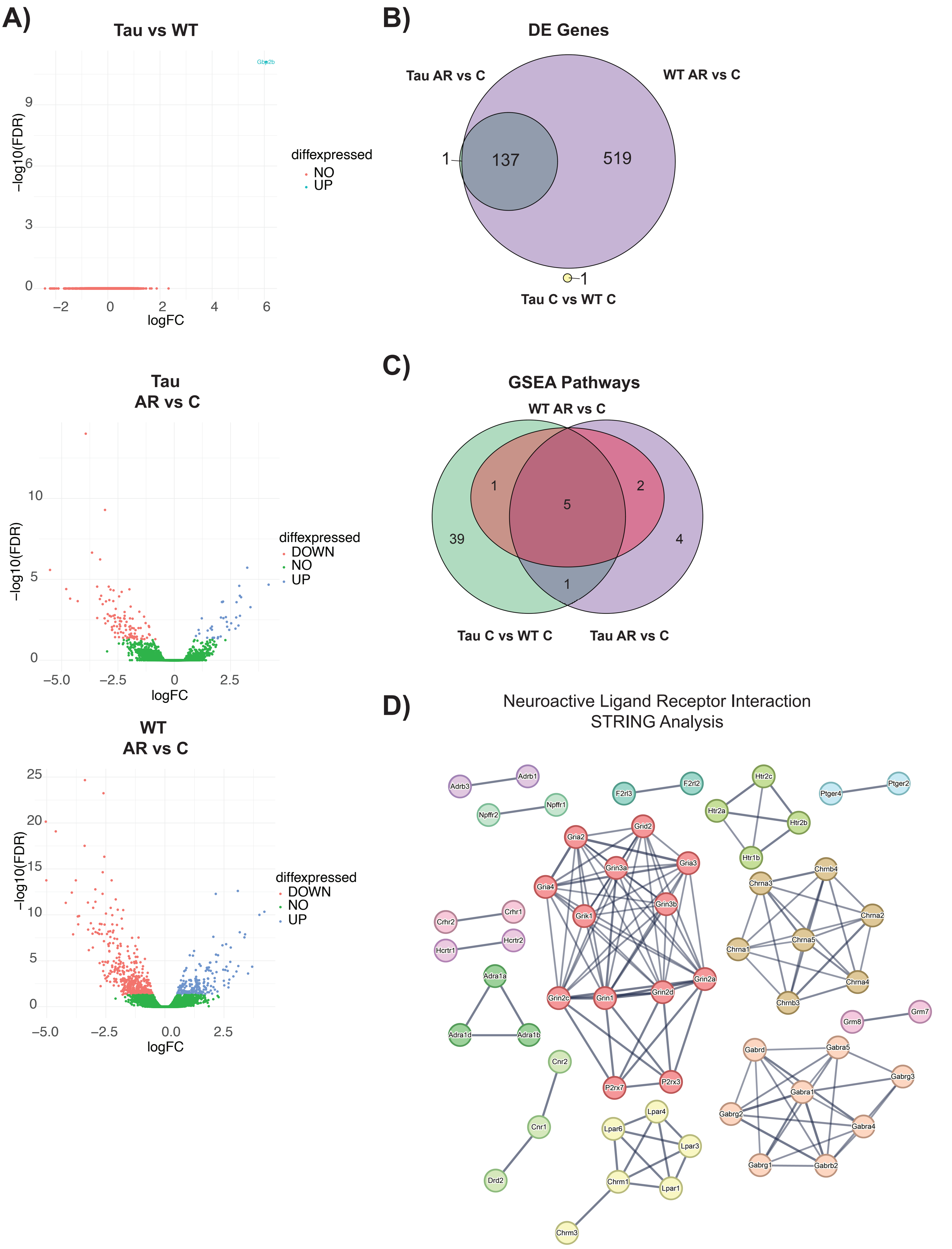

A)
Tau vs WT
Gbp2b
−log10(FDR)
logFC
diffexpressed
NO
UP
B)
DE Genes
Tau AR vs C
WT AR vs C
1
137
519
1
Tau C vs WT C
Tau
AR vs C
diffexpressed
DOWN
NO
UP
C)
GSEA Pathways
WT AR vs C
1
5
2
39
4
1
Tau C vs WT C
Tau AR vs C
WT
AR vs C
D)
Neuroactive Ligand Receptor Interaction
STRING Analysis
Adrb3
Adrb1
Npffr2
Npffr1
F2rl3
F2rl2
Htr2c
Htr2a
Htr2b
Htr1b
Ptger4
Ptger2
Gria2
Grid2
Grin3a
Gria3
Gria4
Grin3b
Grik1
Grin2a
Grin2c
Grin1
Grin2d
P2rx7
P2rx3
Chrnb4
Chrna3
Chrna2
Chrna1
Chrna5
Chrna4
Chrnb3
Crhr2
Crhr1
Hcrtr1
Hcrtr2
Adra1a
Adra1d
Adra1b
Cnr2
Cnr1
Drd2
Lpar4
Lpar6
Lpar3
Chrm1
Lpar1
Chrm3
Grm8
Grm7
Gabrd
Gabra5
Gabrg3
Gabra1
Gabrg2
Gabra4
Gabrg1
Gabrb2
